# Chemical Compounds from Female and Male Rectal Pheromone Glands of the Guava Fruit Fly, *Bactrocera correcta*

**DOI:** 10.3390/insects10030078

**Published:** 2019-03-18

**Authors:** Xiuge Zhang, Chengmei Wei, Jin Miao, Xiaojiao Zhang, Bo Wei, Wenxia Dong, Chun Xiao

**Affiliations:** State Key Laboratory for Conservation and Utilization of Biological Resources, College of Plant Protection, Yunnan Agricultural University, Kunming 650201, Yunnan, China; zxg19890113@163.com (X.Z.); 18487173948@163.com (C.W.); 18487096080@139.com (J.M.); zhangxiaojiaoxwg@163.com (X.Z.); weibo15094267816@163.com (B.W.)

**Keywords:** rectal gland extracts, rectal gland compounds, GC-MS, bioassay, pheromone communication

## Abstract

The guava fruit fly, *Bactrocera correcta*, is one of the major pests affecting mango (*Mangifera indica*) and guava (*Psidium guajava*) production in China. The compound β-caryophyllene was identified from the rectal gland extracts of wild *B. correcta* males and was demonstrated to be a more specific and potent male lure than methyl eugenol (ME) for *B. correcta*. In order to find potential additional pheromone attractants for the monitoring and mass-trapping of this fruit fly, a series of chemical and behavioral assays were conducted in this study. Ten compounds were identified from the rectal glands of virgin *B. correcta* females. These compounds consisted of five major compounds (i.e., ethyl dodecanoate, ethyl tetradecanoate, ethyl *(E)*-9-hexadecenoate, ethyl hexadecanoate, and ethyl *(Z)*-9-octadecenoate) in high quantities, and other compounds (i.e., octanal, N-(3-methylbutyl) acetamide, (*Z*)-9-tricosene, ethyl octadecanoate, and ethyl eicosanoate) in trace amounts, while virtually no compounds were found in male rectal glands. The bioassays indicate that female rectal gland extracts are attractive to virgin females and males. Furthermore, a cyclical production of the five major compounds was found, recurring at roughly 10-d intervals with peaks in 10–13-, 25-, and 35-d-old females. Collectively, these results will contribute to the understanding of pheromone communication in *B. correcta* and may provide important information for improving existing monitoring and control methods for this pest.

## 1. Introduction

The guava fruit fly *Bactrocera correcta* (Bezzi) (Diptera: Tephritidae) is an economically important insect pest that is widely distributed in India, Pakistan, Nepal, Sri Lanka, and Thailand [1], and it has now spread to Burma, Vietnam, and China [2]. With highly adaptive reproductive and dispersal abilities, this fruit fly is considered to be highly invasive and is considered to be a key quarantine pest by many countries [3,4]. *B. correcta* is known to infest more than 60 species of fruits and vegetables in 30 families in tropical and subtropical regions of the world, including guava, mango, cherry, jujube, citrus, and chilli [5]. In China, it causes extensive damage to mango and guava production [6,7].

Today, the control of *B. correcta* mainly depends on the use of chemical insecticides, which affect human health and environmental safety [8]. Methyl eugenol (ME) is a well-known attractant for the monitoring and trapping of populations of *B. correcta* and *B. dorsalis* [9]. However, ME only attracts male flies [10,11], thus limiting the possibility of monitoring the entire fly population or trapping females. To develop new strategies for the detection, monitoring, and trapping of this pest, pheromones that are safer and better attractants need to be developed [12]. It is well known that pheromone components of several *Bactrocera* species, usually released by males, can be isolated and identified by extracting the rectal glands [13,14,15,16,17]. The only exception is the olive fruit fly, *B. oleae*, in which females also produce a multicomponent sex pheromone in the rectal glands. The most abundant compound, 1,7-dioxaspiro [5.5] undecane, has been demonstrated to instigate the highest biological activity in *B. oleae* males, both in the laboratory and in field cage tests [18,19]. 

In *B. correcta*, mating occurs at dusk, and often lasts beyond dusk with a receptive female [19]. During courtship, males perform only brief wing fanning, but females are not attracted to wing fanning males [20]. Therefore, chemical cues (pheromones) released by males or females may play a key role in the attraction of conspecific insects during mate searching. Additionally, age and mating have been shown to affect the quantity of pheromones produced by many insect species [19]. However, research on *B. correcta* pheromones is scarce. Previous studies have shown that laboratory-reared *B. correcta* males from the mass rearing program for the sterile insect technique accumulate large quantities of (Z)-coniferyl alcohol (ZCF) and (Z)-3,4-dimethoxycinnamyl alcohol (DMC) in the rectal glands after feeding on ME [10,21]. However, wild males (that did not feed on ME) were shown to produce large quantities of β-caryophyllene, α-humulene, and alloaromadendrene, along with ZCF and DMC in trace amounts [10]. The compound β-caryophyllene has also been demonstrated to be a more specific and potent male lure than ME for *B. correcta* [11,22]. Currently, however, the chemical composition of rectal pheromone glands of *B. correcta* females has not been investigated, and the age-related chemical composition of both sexes is unknown. In this study, we aimed to identify and characterize the chemical components of the female and male rectal glands of *B. correcta* at different ages. Then, based on the number of compounds identified from female rectal gland extracts, we aimed to investigate the attractiveness of rectal gland extracts from 13-d-old virgin females to conspecific males and females. Finally, we also analyzed age-related changes of the five major compounds found in female rectal glands.

## 2. Material and Methods

### 2.1. Insects

*B. correcta* larvae were originally collected from fallen fruits in the mango orchards of Yuanjiang County, Yunnan Province, China in May 2017. Fruits were transported to the laboratory located in Kunming, Yunnan, and placed in a perspex cage (38 cm × 38 cm × 38 cm) that had a layer of sand on the bottom for pupation. Emerged adult flies were fed on an artificial diet consisting of a mixture of yeast and glucose (ratio 1:5 w/w); water was provided separately in a glass beaker covered with gauze and foam. Mango fruits were provided for female oviposition and larval development. All of the flies were maintained under controlled conditions (25–27 °C, 30–50% relative humidity, and 14:10 h light/dark photoperiod). To obtain virgin females, fourth-generation flies were separated by sex within 24 h of emergence and placed in a nylon gauze cage (15 cm × 15 cm × 15 cm) with water and food until flies reached the appropriate age for pheromone analysis. 

### 2.2. Extraction of Rectal Gland Compounds

Rectal glands were extracted from virgin females and males at 5, 10, 13, 15, 17, 20, 22, 25, 27, 30, 35, and 40 d after birth. For each extraction, five to eight rectal glands were carefully dissected out by pulling off the ovipositor using a pair of forceps under a stereomicroscope (Leica, Wetzlar, Germany), and immediately immersed in 50 μL hexane from 15:00–18:00 h at room temperature. After extraction for 3 h, rectal glands were removed and extracts were stored at −20°C for future analysis. Extractions were performed three times for each age and sex. 

### 2.3. Gas Chromatography-Mass Spectrometry and Gas Chromatography-Flame Ionization Detection

GC-MS analyses were performed on a SHIMADZU GC-MS QP2010 Ultra (Shimadzu, Kyoto, Japan) with a DB-5 capillary column (60 m × 0.32 mm × 0.25 µm) (Agilent Technologies, Santa Clara, CA, USA). Chromatography injections of each rectal sample (1 μL) were made in the split mode configuration (10:1) at 250 °C, and helium was used as the carrier gas at a flow rate of 3.0 mL/min. The initial temperature of the oven was 40 °C, programmed to increase by 5 °C/min up to 260 °C and held at the maximum temperature for 15 min. For mass spectrometry (MS), the ionization voltage was set at 70 eV, the mass range was from 35–500 amu with a scan speed of 1666 amu/s and a scan interval of 0.3 s. The solvent delay time was 3 min. The ionization source and interface temperatures were 230 °C and 250 °C, respectively. Data collection and analyses were performed by using the GC-MS solution software (Shimadzu, Kyoto, Japan) with the mass spectra libraries NIST 14 and NIST 14s (National Institute of Standards and Technology, Mass Spectra Libraries, Gaithersburg, MD, USA). 

Compounds were preliminarily identified by NIST, then identification was confirmed by comparing retention indices with authentic standards, as well as by comparing their retention indices and mass spectra with those found in the NIST Chemistry WebBook [23] and in the literature [24]. Retention indices were calculated by using a gas chromatograph GC 7890A (Agilent Technologies) with a flame ionization detector (FID), equipped with an HP-5 capillary column (30 m × 0.32 mm × 0.25 µm) (Agilent Technologies) under the aforementioned temperature conditions. The retention index (RI) for each compound was calculated by using the following formula: RI = 100Y + 100(Z-Y) × (RT_X_ − RT_Y_)/(RT_Z_ − RT_Y_), where Z and Y represent Z and Y carbon numbers in n-alkanes, RT_X_ is the retention time of an unknown compound, and RT_Z_ and RT_Y_ are the retention times of n-alkanes. 

Gas chromatography-flame ionization detection (GC-FID) was used for the quantification of rectal gland compounds affected by age. Identified compounds were quantified by absolute calibration curves obtained from authentic standards. Based on peak areas, the calibration curves were constructed by injecting a series of 2-μL solutions containing each standard in n-hexane (Merck KGaA, Darmstadt, Germany) into the GC-FID (*n* = 5). One standard solution containing octanal, N-(3-methylbutyl) acetamide, ethyl dodecanoate, ethyl tetradecanoate, ethyl (*E*)-9-hexadecenoate, and ethyl hexadecanoate was made in concentrations of 15, 30, 50, 100, and 150 μg/mL, and the correlation coefficient (*R*^2^) of these standards exceeded 0.99. Another standard solution containing ethyl (*Z*)-9-octadecenoate, ethyl octadecanoate, (*Z*)-9-tricosene, and ethyl eicosanoate was made in concentrations of 15, 30, 50, 60, and 100 μg/mL, and the *R*^2^ of these standards ranged from 0.96–0.98.

### 2.4. Chemicals

Ten standard chemicals were used for the identification and quantification of rectal gland compounds, namely octanal (>98%, TCI, Shanghai, China), N-(3-methylbutyl) acetamide (>95%, Fluorochem, Old Glossop, U.K.), ethyl dodecanoate (>99%, TCI, Shanghai, China), ethyl tetradecanoate (>98%, TCI, Shanghai, China), ethyl (*E*)-9-hexadecenoate (>99%, Nu-chek, Elysian, MN, USA), ethyl hexadecanoate (95%, Fluorochem, Old Glossop, U.K.), ethyl (*Z*)-9-octadecenoate (>99%, Nu-Chek-Prep, Elysian, MN, USA), ethyl octadecanoate (>96%, TCI, Tokyo, Japan), (*Z*)-9-tricosene (88%, J&K, Beijing, China), and ethyl eicosanoate (10 mg/ml in hexane, AccuStandard, New Haven, CT, USA).

### 2.5. Bioassays

A glass Y-tube olfactometer was used to test the attractiveness of female *B. correcta* rectal gland extracts of conspecific virgin females and males at 15–16 days old. The olfactometer consisted of a main tube (13 cm long, 2 cm diameter) with two arms (13 cm long) configured at a 30° angle. Each arm was connected to a glass adaptor (9 cm long, 1 cm diameter) as an odor source for the treatment or the control. Rectal gland extracts of 13-d-old females (when female gonad is completely mature) were tested against a hexane (control); extracts of rectal glands were prepared as described above. Next, 10 μL of extracts (corresponding to one gland) were adsorbed on filter paper (0.5 cm × 5 cm) and the solvent was evaporated for 30 s. Treated filter paper was then placed into the treatment adaptor by insertion into an olfactometer; filter paper treated with 10 μL of hexane was placed into the control adaptor. Charcoal-purified humidified air was transmitted through the arms at 10 mL/min to carry the stimuli to the test insects. A fly was introduced individually in the main arm of the Y-tube using a glass vial and observed for 3 min. The choice of a given cue was recorded when the fly exhibited a searching behavior at the end of the main arm, then moved towards the cue in one of the arms of the Y-tube olfactometer and remained there for at least 30 s. If a fly remained in the main arm but did not make a choice within 3 min, data was recorded as “no choice”. After five replications, the filter paper was replaced, and the olfactometer was cleaned with ethanol (>99.7%, Westlong Chemical Co., Ltd, Tianjin, China), then dried at 160 °C for 30 min. After two repetitions, the position of the arms of the Y-tube olfactometer was alternated to avoid positional effect. Each fly was tested once, and a total of 42 replications with responsive flies were performed. The room was lit by vertically hanging daylight fluorescent tubes (30 W) (Philips, Shanghai, China), and the light intensity on the arms of the olfactometer was approximately 300 lux. All of the experiments were conducted between 16:30–20:00 h in an environmentally controlled room (27 ± 2 °C, 40 ± 10% RH). 

### 2.6. Statistical Analysis

A chi-square test with Yates’s correction was conducted to evaluate the differences between the number of *B. correcta* males and females entering each arm of the olfactometer.

## 3. Results

### 3.1. Chemical Identification and Quantification of Rectal Gland Compounds

A total of 10 compounds were identified from the n-hexane extracts of *B. correcta* female and male rectal glands (Table 1). In female rectal glands, 10 compounds were identified. Of these compounds, six were present in female rectal glands at different ages (i.e., ethyl dodecanoate, ethyl tetradecanoate, ethyl (*E*)-9-hexadecenoate, ethyl hexadecanoate, ethyl (*Z*)-9-octadecenoate, and ethyl octadecanoate) (see Table S1). There were five compounds (i.e., ethyl dodecanoate, ethyl tetradecanoate, ethyl (*E*)-9-hexadecenoate, ethyl hexadecanoate, and ethyl (*Z*)-9-octadecenoate) found in amounts higher than 50 ng/gland at almost every age tested and these were thus classified as major compounds. Ethyl octadecanoate was found in amounts lower than 50 ng/gland and was thus classified as a minor compound. The other four compounds (i.e., octanal, N-(3-methylbutyl) acetamide, (*Z*)-9-tricosene, and ethyl eicosanoate) were found in amounts lower than 60 ng/gland and were not always present in females at each age tested (see Table S1 in the supplementary materials). In male rectal glands, nine compounds were identified in negligible amounts (<50 ng/gland) at almost all ages tested and were produced unevenly by males at the same ages as females (see Table S1 in the supplementary materials).

### 3.2. Bioassays

**Figure 1:**
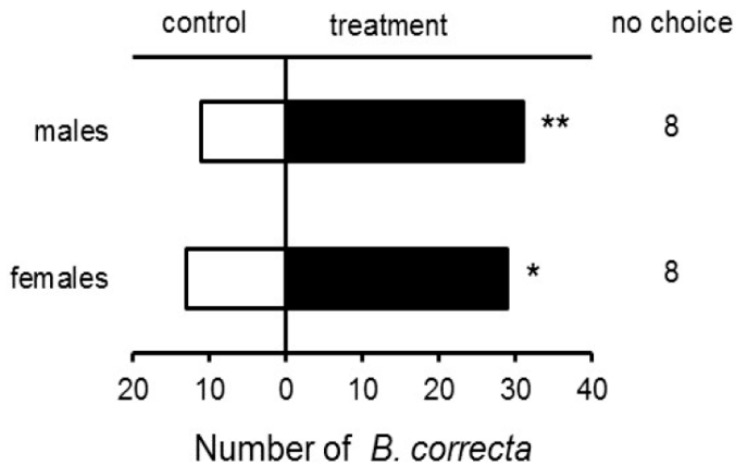
Responses of male and female *B. correcta* to rectal gland extracts of 13-d-old virgin females vs. hexane (control), where 50 flies were tested; * indicates significant differences at *p* < 0.05 and ** indicates *p* < 0.01, respectively, per chi-square test and Yates’s correction.

Both *B. correcta* virgin females (*χ*^2^ = 5.3571, *p* = 0.0206) and males (*χ*^2^ = 8.5952, *p* = 0.00337) were significantly more attracted to female rectal gland extracts than the control (Figure 1). 

### 3.3. Age-Related Changes in the Major Compounds Found in Female Rectal Pheromone Glands

**Figure 2:**
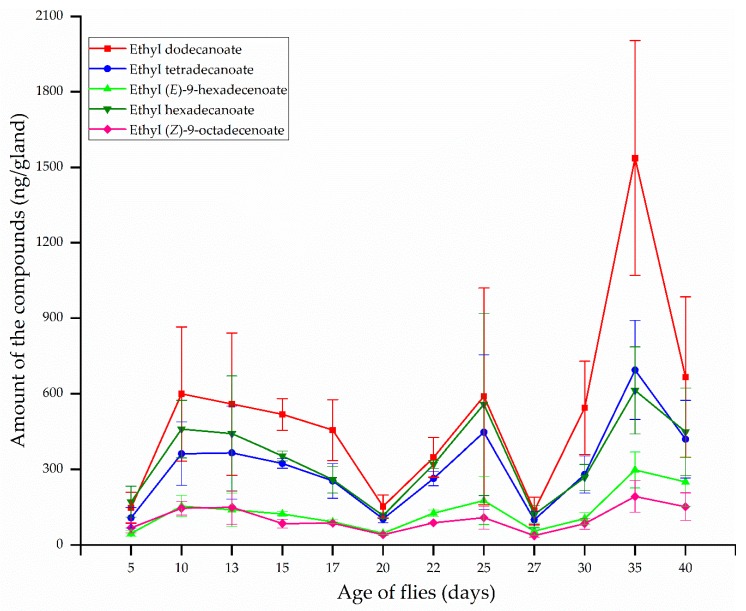
Changes in rectal gland compound quantities, depending on age, from *B. correcta* females (mean ± standard error).

The most abundant compounds in the rectal glands of females at all ages were ethyl dodecanoate, ethyl hexadecanoate, and ethyl tetradecanoate, followed by ethyl (*E*)-9-hexadecenoate and ethyl (*Z*)-9-octadecenoate (Figure 2). The amount of these compounds varied with age and reached maximum quantity at 10–13 d old when females produced large quantities of ethyl dodecanoate (599.59 ng/gland), ethyl tetradecanoate (365.92 ng/gland), ethyl (*E*)-9-hexadecenoate (154.66 ng/gland), ethyl hexadecanoate (459.91 ng/gland), and ethyl (*Z*)-9-octadecenoate (149.92 ng/gland). The amounts of ethyl dodecanoate and ethyl hexadecanoate decreased by approximately 4 times the maximum quantity to 153.34 ng/gland and 117.28 ng/gland, respectively, at a slow rate until 20 d after emergence. Similarly, the quantities of ethyl tetradecanoate, ethyl (*E*)-9-hexadecenoate, and ethyl (*Z*)-9-octadecenoate decreased by approximately 3.5 times the maximum quantity to 104.07, 47.01, and 40.81 ng/gland, respectively. Subsequently, the amounts of the five compounds increased again to their maximum quantities of 590.02, 447.40, 176.46, 557.92, and 108.76 ng/gland, respectively, at 25 d after emergence. Then, these compounds decreased dramatically until 27 d old, thereafter increasing to their maximum quantities at 35 d. After emergence at 35 d, maximum quantities of these compounds were 1536.94, 694.27, 298.24, 613.25, and 193.19 ng/gland, respectively, and were greater than quantities at 10–13 and 25 d after emergence (Figure 2). 

## 4. Discussion

In this study, we identified and evaluated the compounds found in the rectal glands of virgin female and male *B. correcta*. Chemical analyses revealed that the compounds of male rectal glands were produced in extremely low quantities. In a previous study, Tokushima et al. (2010) demonstrated that wild *B. correcta* males accumulated large quantities of β-caryophyllene, α-humulene, and alloaromadendrene, as well as trace amounts of ZCF and DMC in the rectal pheromone gland; these compounds were isolated by extracting the rectal glands of wild males with ethanol [10]. Furthermore, laboratory-raised males were demonstrated to also sequester β-caryophyllene and α-humulene, along with ZCF and DMC, after feeding on a mixture of ME, β-caryophyllene, and α-humulene [10]. Notably, β-caryophyllene and α-humulene are common volatile constituents in mango and guava fruits [25,26,27,28]. Therefore, because negligible amounts of these compounds were detected in our laboratory-reared males, which were fed an artificial diet during adult stages, we speculate that adult males may need to consume host fruit volatiles then store them, or their analogues or metabolites, in their rectal glands. However, in other *B. dorsalis* complex species, males without ME (or other compounds) feeding produced high quantities of rectal gland secretions [13,14,15]. This difference may confirm that *B. correcta* is outside of the *B. dorsalis* complex [21]. Additionally, our results verify the presence of the 10 compounds identified in the rectal glands of virgin *B. correcta* females. These compounds consist of five major compounds in high quantities and five compounds in trace amounts. Thus, we suggest that pheromone components may be present in female rectal glands. Moreover, in this study, bioassays were conducted to determine if female rectal glands were attractive to conspecific female and male flies, and our results revealed that both virgin females and males were significantly more attracted to female rectal gland extracts than the control (hexane). Similar results were found in the Chinese citrus fly, *B. minax*, such that female rectal gland extracts were attractive to male and female adults in the field [29]. The results suggest that some of the components of female rectal gland extracts may be involved in intraspecific communication, and imply the presence of pheromone compounds in *B. correcta* female rectal glands. 

Our results revealed that the five major compounds (i.e., ethyl dodecanoate, ethyl tetradecanoate, ethyl (*E*)-9-hexadecenoate, ethyl hexadecanoate, and ethyl (*Z*)-9-octadecenoate) were produced in *B. correcta* female rectal glands at all ages. Interestingly, four of these compounds (i.e., ethyl dodecanoate, ethyl tetradecanoate, ethyl hexadecanoate, and ethyl (*Z*)-9-octadecenoate) have also been identified in the rectal glands of virgin *B. oleae* females, but conspecific attraction was not observed [30]. In contrast, methyl hexadecanoate and ethyl decanoate has been identified in the rectal glands of virgin *B. oleae* females and attracted conspecific males and females [30]. Furthermore, these compounds were recently found in the cuticular hexane body washes of females from *B. carambolae* and *B. dorsalis* (*B. dorsalis*, *B. papayae*, *B. philippinensis* and *B. invadens*), but the function of these compounds in these species’ females has not been determined [31]. As for the compound ethyl (*E*)-9-hexadecenoate, although it was different from compounds that have been reported in the literature, it is structurally very close to identified compounds. For instance, Canale et al. (2015) identified methyl (*Z*)-9-hexadecenoate in *B. oleae* female rectal glands [30], while Vaníčková et al. (2017) identified ethyl (*Z*)-9-hexadecenoate in the cuticular washes of *B. carambolae* and *B. dorsalis* [31]. Thus, we speculate that the major compounds may have a possible pheromone functional role in *B. correcta*, but further investigation is required.

As for the compounds found in trace amounts in females, our results revealed that octanal, N-(3-methylbutyl) acetamide, (*Z*)-9-tricosene, and ethyl eicosanoate were not always present at different ages, and it appears that these compounds were produced randomly, which also requires further investigation. Meanwhile, ethyl octadecanoate was produced by females at all ages. Among these compounds, N-(3-methylbutyl) acetamide was identified in male rectal gland extracts and has been reported as a pheromone volatile in *B. tryoni* [17,32], and has been found to be attractive to *B. carambolae* females [11,14]. Additionally, N-(3-methylbutyl) acetamide was found in volatiles from *B. tryoni* females, but their functional role is not known [33]. A separate sex pheromone, (Z)-9-tricosene, of the female house fly, *Musca domestica* (L.) [34,35], was first detected only in the rectal gland extracts of *B. oleae* males [36] and was found to be the main male sex pheromone component for female attraction [33]. As for two other minor compounds, ethyl octadecanoate was identified from rectal gland extracts in *B. oleae* females [37], and ethyl eicosanoate was identified from cuticular body washes in the females of *B. carambolae* and four species of the *B. dorsalis* complex [38]. Therefore, we speculate that the presence of these minor compounds may be responsible for attracting *B. correcta* females and males to some extent. However, the exact pheromone function will require further investigation.

In order to help identify potential pheromone components, as well as provide some information related to mate choice, our study also investigated age-related changes in five major compounds in the rectal pheromone glands of *B. correcta* females. It appeared that there was a cyclical production of the major compounds, recurring on roughly 10-d intervals with peaks in 10–13-, 25-, and 35-d-old females, which is similar to the production of 1,7-dioxaspiro [5.5] undecane in the pheromone gland extracts of 1–31-d-old *B. oleae* females [19]. Thus, it appears that the first production and emission periods of these compounds were 5–10 and 13–20 d after emergence. Thereafter, two more production and emission periods were observed until 40 d after emergence. Therefore, we speculate that age-dependent quantitative changes of these major compounds may be related to pheromone production and emission in females, thereby affecting courtship and mating in *B. correcta*. This implies that all of the five compounds in female rectal glands of *B. correcta* may play a role in mate selection. Additionally, at 25 d, the quantities of ethyl tetradecanoate, ethyl (*E*)-9-hexadecenoate, and ethyl hexadecanoate were higher than at 10–13 d. At 35 d after emergence, five major compounds were found in quantities higher than that at 10–13 and 25 d. In other words, the quantities of these compounds produced by virgin females during the first production period were lower than the other two production periods occurring 20–40 d after emergence. Mazomenos (1984) demonstrated that the quantity of 1,7-dioxaspiro [5.5] undecane produced by mated *B. oleae* females was lower than that produced by virgin females at the same age [19], thus, we speculate that the mating status of *B. correcta* females may influence the production of the five major compounds in their rectal glands. This also implies that the five major compounds in *B. correcta* female rectal glands may play a role in mate selection.

## 5. Conclusions

In conclusion, our results demonstrate for the first time that large quantities of rectal gland compounds were found in *B. correcta* females, while *B. correcta* males produced only negligible amounts of the same compounds in their rectal glands. The bioassays also reveal that *B. correcta* female rectal gland extracts were attractive to both virgin females and males, thus, we speculate that the pheromone compounds may be present in female rectal gland extracts. Furthermore, we investigated the production of the five major compounds in rectal glands from *B. correcta* females, which were found to vary with age. The findings of this study reveal that the age of the fly may be important for mate choice, and the results may be used to help identify potential pheromone components involved in the mating process. Overall, these results contribute to the understanding of the pheromone communication system in *B. correcta* and may provide important information that could improve existing monitoring and control methods. However, further research is required in order to investigate these compounds found in female rectal glands and their attractiveness to conspecific females or males.

## Figures and Tables

**Table 1:** Chemical compounds identified by GC-MS of rectal glands in female and male *B. correcta.*

Peak	Compound	RI	RI_ref_	MS Data [m/z (%)]
1	Octanal	1002	999	100 (10), 84 (58), 69 (45), 67 (25), 57 (72), 56 (74), 55 (49), 43 (100), 41 (81)
2	N-(3-Methylbutyl) acetamide	1159	-	129 (6), 114 (16), 86 (28), 73 (100), 72 (78), 70 (10), 60 (37), 55 (21), 44 (53), 43 (77), 41 (20)
3	Ethyl dodecanoate	1599	1595	228 (4), 183 (12), 157 (13), 101 (49), 88 (100), 73 (21), 70 (24), 61 (14), 55 (21), 43 (21), 41 (19)
4	Ethyl tetradecanoate	1798	1796	256 (6), 213 (13), 157 (17), 101 (53), 89 (14), 88 (100), 73 (20), 70 (22), 55 (23), 43 (24), 41 (20)
5	Ethyl (*E*)-9-hexadecenoate	1973	1977	282 (5), 101 (53), 98 (47), 97 (60), 96 (56), 88 (68), 84 (52), 83 (60), 69 (78), 55 (100), 41 (53)
6	Ethyl hexadecanoate	1996	1993	284 (8), 157 (17), 101 (56), 89 (16), 88 (100), 73 (18), 70 (21), 57 (20), 55 (26), 43 (28), 41 (21)
7	Ethyl (*Z*)-9-octadecenoate	2169	2169	310 (4), 101 (37), 98 (35), 97 (49), 96 (42), 88 (49), 84 (39), 83 (53), 69 (66), 55 (100), 41 (56)
8	Ethyl octadecanoate	2197	2197	312 (11), 157 (21), 101 (59), 89 (18), 88 (100), 73 (18), 70 (20), 57 (24), 55 (28), 43 (33), 41 (22)
9	(*Z*)-9-Tricosene	2296	2298	322 (2), 139 (12), 125 (25), 111 (43), 97 (83), 83 (90), 69(82), 57 (100), 55 (92), 43 (76),41 (55)
10	Ethyl eicosanoate	2399	2400	340 (16), 157 (21), 101 (60), 89 (22), 88 (100), 73 (16), 70 (20), 57 (27), 55 (29), 43 (35), 41 (21)

RI is the retention index on HP-5, and RI_ref_ is the retention index obtained from the National Institute of Standards and Technology (NIST) Chemistry WebBook [23] and the literature [24].

## Supplementary Materials

The following are available online at https://www.mdpi.com/2075-4450/10/3/78/s1, Table S1: Chemical compounds identified by GC-MS in rectal glands of *B. correcta* females and males at different ages.
