# Supplementary material for: Chemical Compounds from Female and Male Rectal Pheromone Glands of the Guava Fruit Fly, Bactrocera correcta
Source: Insects. 2019 Mar 18;10(3):78. doi: 10.3390/insects10030078 (PMC6468847; doi:10.3390/insects10030078)
Supplement: Supplementary file 1 [file insects-10-00078-s001.pdf]

Table S1. Chemical compounds identified by GC-MS in rectal glands of *B. correcta* females and males at different ages.

Age (day-olds)	Insect	Amounts (ng/gland)									
		A	B	C	D	E	F	G	H	I	J
5	F	-	-	148.03 ± 61.23	108.4 ± 40.54	46.12 ± 13.39	171.79 ± 61.50	68.58 ± 20.70	28.63 ± 6.88	-	-
	M	-	32.60 ± 13.89	-	-	23.70 (1)	31.30 ± 6.63	46.27 ± 12.55	48.96 (1)	21.44 (1)	48.72 ± 2.94
10	F	-	44.72 ± 23.19	599.59±265.67	362.47±125.50	154.66±42.45	459.91 ± 113.97	145.93 ± 27.37	43.52 ± 6.60	40.16 ± 24.13	-
	M	-	-	-	-	-	17.53 (1)	-	-	48.69 (1)	49.07 (1)
13	F	-	-	558.99 ± 282.38	365.92 ±184.06	140.19 ± 65.92	441.31 ±229.29	149.92 ± 67.74	38.88 ± 14.07	29.13±9.61	-
	M	15.69 (1)	-	-	13.62 (1)	-	25.53 (1)	-	20.04 (1)	41.10 (1)	20.56 (1)
15	F	-	33.80 ± 4.28	518.14 ± 62.91	323.84 ± 19.28	123.76 ± 8.18	353.33 ± 20.10	85.56 ± 17.54	29.80 ± 0.79	-	-
	M	-	-	-	25.13 (1)	31.56 (1)	66.66 ± 3.48 (1)	-	57.32 ± 30.15	27.44 (1)	-
17	F	30.59 ± 0.01	58.20 ± 26.66	455.88 ± 120.80	254.81 ± 68.68	92.14 ± 7.58	259.67 ± 53.02	86.65 ± 4.09	39.33 ± 9.21	-	-
	M	-	-	-	-	-	28.17 (1)	-	23.61 (1)	11.05 (1)	-
20	F	-	-	153.34 ± 45.86	104.08 ± 15.58	47.01 ± 6.02	117.28 ± 12.80	40.81 ± 3.10	-	-	-
	M	-	-	-	-	-	-	-	-	-	-
22	F	-	-	347.54 ± 78.30	264.31 ± 28.82	126.29 ± 13.40	320.07 ± 15.62	88.67 ± 1.30	34.55 ± 3.76	33.18 ± 3.61	33.97 ± 8.50
	M	-	-	-	14.27 (1)	-	16.42 (1)	-	-	-	-
25	F	-	-	590.02 ± 429.96	447.40 ± 306.55	176.46 ± 95.33	557.92 ± 361.53	108.76 ± 45.16	39.97 ± 4.70	-	-
	M	9.26 (1)	-	-	-	-	14.09 (1)	-	-	-	-
27	F	-	-	135.91 ± 54.86	100.45 ± 31.63	55.33 ± 15.60	121.48 ± 34.76	37.46 ± 6.24	14.61 ± 1.90	-	-
	M	-	-	-	-	-	-	-	-	-	-
30	F	-	35.38 ± 7.30	544.05 ± 185.17	280.62 ± 73.71	105.41 ± 22.66	268.70 ± 51.32	84.68 ± 22.11	30.09 ± 4.39	-	-
	M	-	-	-	-	-	14.08 (1)	-	-	-	-
35	F	-	-	1536.94 ± 466.55	694.27 ± 196.44	298.24 ± 71.57	613.25 ± 172.84	193.19 ± 63.17	45.00 ± 8.38	39.68 ± 5.32	41.35 ± 6.79
	M	9.26 (1)	-		-	-	-	-	-	-	-

				-							
40	F	-	-	666.30 ± 318.56	419.66 ± 153.49	250.78 ± 97.07	448.72 ± 173.74	152.30 ± 54.96	46.37 ± 9.86	43.23 ± 11.11	43.92 ± 18.04
	M	22.97 ± 0.20	43.78 (1)	-	-	25.76 (1)	-	-	-	-	-

F females; M males. A octanal; B N-(3-methylbutyl) acetamide; C ethyl dodecanoate; D ethyl tetradecanoate; E ethyl (*E*)-9-hexadecenoate; F ethyl hexadecanoate; G ethyl (*Z*)-9-octadecenoate; H ethyl octadecanoate; I (*Z*)-9-tricosene and J ethyl eicosanoate. Number 1 in parentheses represents only one of three replicates where the compound was detected.
